# Supplementary material for: Health workers and Sub Saharan African women’s understanding of equal access to healthcare in Norway
Source: PLoS One. 2021 Sep 10;16(9):e0255934. doi: 10.1371/journal.pone.0255934 (PMC8432872; doi:10.1371/journal.pone.0255934)
Supplement: S1 Appendix — (DOCX) [file pone.0255934.s001.docx]

S1 Appendix. This is the interview guide for health-personnel

Topics to be investigated, discussed and clarified

.

Information about the institution

- - Location, number of staff, what staff, number of patients, patient categories
  - Experiences with patients suffering from FGM/C
  - Experiences with patterns of mental health in different categories of population, immigrant women generally and women suffering from FGM/C or SSA women
  - Descriptions of the structure of referrals

Competence on FGM/C

- - What competence on FGM/C does the staff have?
  - Types of FGM/C, prevalence in different countries, within different ethnic groups
  - Consequences of FGM/C, physical and psychological consequences, short and long term
  - Competence and experiences with patients with FGM/C and treatments, duration and follow-ups

Patients

- Who are the patients that has problems with FGM/C
- Describe patterns of patients’ needs when suffering mental consequences of FGM/C
- Describe patterns of patients’ needs in general for SSA women
- Experiences of socio-sexual problems
- How do patients communicate their problems
- Challenges – diagnosis
- Expectations – disappointments – satisfactions
- Alternative treatment – home travel
- Somatization of mental health problems
- Referrals where

Thresholds, time, cost, availability

- Patterns of rejection and inclusions.
- How serious must the problem be for being referred and accepted
- Sign of mental health problems due to FGM/C
- The repertoire of health care for cut patients
- Communication and use of interpreters
- Alternatives for referrals –flow of referrals –challenges. Who goes where?
- The role of the municipality
- Who pays the treatment

Consultations

- What happens within a consultation
- Interaction
- Challenges
- Conduct understanding and challenges
- Feed back
- Other

Equal access to health care

- - Do African women get equal access to health care as ethnic Norwegians
  - Describe reasons why they get, or do not get equal access
