# Supplementary material for: Health workers and Sub Saharan African women’s understanding of equal access to healthcare in Norway
Source: PLoS One. 2021 Sep 10;16(9):e0255934. doi: 10.1371/journal.pone.0255934 (PMC8432872; doi:10.1371/journal.pone.0255934)
Supplement: S2 Appendix — (DOCX) [file pone.0255934.s002.docx]

S2 Appendix. This is interview guide for SSA women

1. Personal information
   1. City or municipality of residence
   2. Gender
   3. Age
   4. Married/unmarried/single
   5. Occupation/education
   6. Religion
   7. Years of residence in Norway
   8. Country of birth
2. Household and network
   1. Amount of household members (age i.e.)
   2. Decisions about seeking healthcare (Who takes them - seriousness of problem, places to go: in and outside the country i.e.)
   3. Seeking advice and information outside the household (from where (other countries) and from who)
3. Healthcare in general
   1. Where do you go when you have health problems (GP –Emergency-Specialist services)
   2. Use of health services – experience – challenges in the use of health care services
   3. Last time when you or your child was ill, what was the problem, and where did you go
   4. Thoughts about healthcare in Norway and original country
4. FGM
   1. Describe your cutting experience (age, ritual, complications, help)
   2. Links between FGM and health problems
   3. Pain
5. Experience
   1. Assessments of the health care services
   2. Description of health problems
   3. Alternative help from healers
   4. Assessments, thoughts, decisions
   5. Challenges
6. Your thoughts about equal access to health care
   1. Is there discrimination – have you experienced it
   2. Challenges
   3. Improvements
   4. Trust
