# Supplementary material for: Health workers and Sub Saharan African women’s understanding of equal access to healthcare in Norway
Source: PLoS One. 2021 Sep 10;16(9):e0255934. doi: 10.1371/journal.pone.0255934 (PMC8432872; doi:10.1371/journal.pone.0255934)
Supplement: Attachment — Submitted filename: PONE-D-20-39751 Comments.pdf [file pone.0255934.s003.pdf]

PLOS ONE

Unequal access to healthcare: a hysteresis condition?

Health workers' and Sub-Saharan African women's understanding of equal access to healthcare in Norway

--Manuscript Draft--

**Manuscript Number:**	PONE-D-20-39751
**Article Type:**	Research Article
**Full Title:**	Unequal access to healthcare: a hysteresis condition?<br><br>Health workers' and Sub-Saharan African women's understanding of equal access to healthcare in Norway
**Short Title:**	Unequal access to healthcare: a hysteresis condition?
**Corresponding Author:**	Inger-Lise Lien, Ph.D<br>Norwegian Centre for Violence and Traumatic Stress Studies<br>Oslo, NORWAY
**Keywords:**	Access to health care, Sub-Saharan African women, SSA-women, Health care workers, Female Genital Mutilation, Cutting, circumcision, unexplained pain, candidacy theory, Bourdieus theory of practice, habitus, bio-medical field, hysteresis, equal, unequal,
**Abstract:**	Aim and Method : This article discuss and analyses equal access to health care, based on interviews with health workers as well as Sub Saharan African Women. The study is conducted in Oslo, Norway, using a qualitative fieldwork research design. One hundred interviews were done with health workers and 55 interviews were done with Somali, Gambian and Eritrean women with the use of a semi structural guideline and purposeful sampling. Results: The study found a mismatch in the conception of equal access to health care between health workers and the SSA-women. On the overall abstract level the health workers held that there was no equal access of health care for SSA-women, while SSA-women argued that there is equal access. However, at the practical level there was a great deal of match between the sides in conceptions of problems in the structure of health care like: little time available to explain and identify symptoms, navigation in the system, permeability like difficulties of getting referrals, adjudication by the health workers i.e. Theory and analysis : The analysis applied Bourdieu's concepts of field, habitus and hysteresis, as well as candidacy theory and concluded that health workers and SSA-women based their experiences of health care within different types of habitus as their baseline for understanding. The SSA-women's habitus is in hysteresis with the current bio medical field, as they seem to base their understanding on earlier experiences from the home country. Theoretical conclusion is that Bourdieu's theory is complementary to the candidacy theory and may function as a meta- theoretical framework for dimensions within the candidacy theory, thereby adding to a deeper understanding of reasons for match, mismatch and hysteresis that was expressed about equal access to health care by the two sides. Another finding was that SSA-women often suffer from unexplained pain, which leads them to seek health care from the religious realm within the traditional habitus, when they do not get satisfactory explanations from the bio medical field in Norway.
**Order of Authors:**	Inger-Lise Lien, Ph.D
**Opposed Reviewers:**	Sara Johnsdotter, Ph.d<br>Professor, Society of Public Health: Royal Society for Public Health<br>sara.johnsdotter@mau.se<br>She has worked on FGM, and published extensively for many years
	Bettina Shell Duncan, Ph.d<br>Professor, University of Washington Natural Sciences Library: University of Washington<br>bsd@uw.edu<br>She has written on Global Health

Powered by Editorial Manager® and ProduXion Manager® from Aries Systems Corporation

	Gareth Wiltshire, Ph.d<br>Lecturer, Loughborough University<br>G.Wiltshire@lboro.ac.uk<br>He uses Bourdieus theories on habitus and field in studies of health.
	Mary Dixon-Woods, Ph.d<br>Professor and director, John Hopkins University<br>director@thisinstitute.cam.ac.uk<br>She developed the candidacy theory used in this paper
**Additional Information:**	
**Question**	**Response**
**Financial Disclosure**<br><br>Enter a financial disclosure statement that describes the sources of funding for the work included in this submission. Review the submission guidelines for detailed requirements. View published research articles from *PLOS ONE* for specific examples.<br><br>This statement is required for submission and **will appear in the published article** if the submission is accepted. Please make sure it is accurate.<br><br>**Unfunded studies**<br>Enter: *The author(s) received no specific funding for this work.*<br><br>**Funded studies**<br>Enter a statement with the following details:<br>• Initials of the authors who received each award<br>• Grant numbers awarded to each author<br>• The full name of each funder<br>• URL of each funder website<br>• Did the sponsors or funders play any role in the study design, data collection and analysis, decision to publish, or preparation of the manuscript?<br>• **NO** - Include this sentence at the end of your statement: *The funders had no role in study design, data collection and analysis, decision to publish, or preparation of the manuscript.*<br>• **YES** - Specify the role(s) played.<br><br>* typeset	The Norwegian Research Council has financed the main part of the research. NKVTS with support from the Ministry of Justice has contributed to the financing of the research.<br>No - The funders had no role in study design, data collection and analysis, decision to publish, or preparation of the manuscript.
**Competing Interests**<br><br>Use the instructions below to enter a competing interest statement for this	The author have declared that no competing interests exist.

submission. On behalf of all authors, disclose any competing interests that could be perceived to bias this work—acknowledging all financial support and any other relevant financial or non-financial competing interests.<br><br>This statement **will appear in the published article** if the submission is accepted. Please make sure it is accurate. View published research articles from *PLOS ONE* for specific examples.<br><br>**NO authors have competing interests**<br><br>Enter: *The authors have declared that no competing interests exist.*<br><br>**Authors with competing interests**<br><br>Enter competing interest details beginning with this statement:<br><br>*I have read the journal's policy and the authors of this manuscript have the following competing interests: [insert competing interests here]*<br><br>* typeset	
**Ethics Statement**<br><br>Enter an ethics statement for this submission. This statement is required if the study involved:<br><br>• Human participants<br>• Human specimens or tissue<br>• Vertebrate animals or cephalopods<br>• Vertebrate embryos or tissues<br>• Field research<br><br>Write "N/A" if the submission does not require an ethics statement.<br><br>General guidance is provided below. Consult the submission guidelines for detailed instructions. **Make sure that all information entered here is included in the Methods section of the manuscript.**	The project is based on interviews with health workers and SSA-women that have been given written<br>and oral information about the project. Informants have signed a written consent.<br>These written documents have been cleared by the Ethics committee.<br>Data has been presented in the manuscript and analysed anonymously.<br>The project got ethical clearance from the Regional Committee for Medical and Health Research Ethics in Norway, that granted permission the 12.09.2017. It has the number 2017/977.<br>https://helseforskning.etikkom.no/forside?_ikbLanguageCode=us<br>All necessary permits were obtained for the described study, which complied with all relevant regulations.

Powered by Editorial Manager® and ProduXion Manager® from Aries Systems Corporation

**Format for specific study types**<br><br>**Human Subject Research (involving human participants and/or tissue)**<br>• Give the name of the institutional review board or ethics committee that approved the study<br>• Include the approval number and/or a statement indicating approval of this research<br>• Indicate the form of consent obtained (written/oral) or the reason that consent was not obtained (e.g. the data were analyzed anonymously)<br><br>**Animal Research (involving vertebrate animals, embryos or tissues)**<br>• Provide the name of the Institutional Animal Care and Use Committee (IACUC) or other relevant ethics board that reviewed the study protocol, and indicate whether they approved this research or granted a formal waiver of ethical approval<br>• Include an approval number if one was obtained<br>• If the study involved *non-human primates*, add *additional details* about animal welfare and steps taken to ameliorate suffering<br>• If anesthesia, euthanasia, or any kind of animal sacrifice is part of the study, include briefly which substances and/or methods were applied<br><br>**Field Research**<br><br>Include the following details if this study involves the collection of plant, animal, or other materials from a natural setting:<br>• Field permit number<br>• Name of the institution or relevant body that granted permission	
**Data Availability**<br><br>Authors are required to make all data underlying the findings described fully available, without restriction, and from the time of publication. PLOS allows rare exceptions to address legal and ethical concerns. See the PLOS Data Policy and FAQ for detailed information.	No - some restrictions will apply

A Data Availability Statement describing where the data can be found is required at submission. Your answers to this question constitute the Data Availability Statement and **will be published in the article**, if accepted.<br><br>**Important:** Stating 'data available on request from the author' is not sufficient. If your data are only available upon request, select 'No' for the first question and explain your exceptional situation in the text box.<br><br>Do the authors confirm that all data underlying the findings described in their manuscript are fully available without restriction?	
**Describe where the data may be found in full sentences. If you are copying our sample text, replace any instances of XXX with the appropriate details.**<br><br>• If the data are **held or will be held in a public repository**, include URLs, accession numbers or DOIs. If this information will only be available after acceptance, indicate this by ticking the box below. For example: *All XXX files are available from the XXX database (accession number(s) XXX, XXX.*).<br>• If the data are all contained **within the manuscript and/or Supporting Information files**, enter the following: *All relevant data are within the manuscript and its Supporting Information files.*<br>• If neither of these applies but you are able to provide **details of access elsewhere**, with or without limitations, please do so. For example:<br><br>*Data cannot be shared publicly because of [XXX]. Data are available from the XXX Institutional Data Access / Ethics Committee (contact via XXX) for researchers who meet the criteria for access to confidential data.*<br><br>*The data underlying the results presented in the study are available*	All relevant data are within the manuscript and its supporting files.

from (include the name of the third party and contact information or URL).<br>• This text is appropriate if the data are owned by a third party and authors do not have permission to share the data.<br><br>* typeset	
Additional data availability information:	Tick here if the URLs/accession numbers/DOIs will be available only after acceptance of the manuscript for publication so that we can ensure their inclusion before publication.

Powered by Editorial Manager® and ProduXion Manager® from Aries Systems Corporation

Dear Editor in PLOS ONE. 04 January 2021

Earlier this year I submitted an article that was published in your journal: Lien/Hertzberg: A system analyses of the mental health services in Norway and its availability to women with female genital mutilation. It was publish the 4th of November 2020: https://doi.org/10.1371/journal.pone.0241194

In addition, I have written a new article describing how health workers and SSA-women in Norway understand equal access to health care. This article I will submit today is a follow up on the previous article. It contains information about equal access from both sides: the supply and the demand side of health care. Pierre Bourdieu's theory of practice is used to understand the mismatch and gaps that was found in the assessments of access between the two sides. Candidacy theory is also used at the lower level to understand what is implied within the dimensions described in this theory of candidacy as a joint negotiation of health care.

The article has 8937 words

I hope your journal will find the article interesting and thereby will be willing to publish it.

I have suggested several reviewers for this paper.

Best regards from Inger-Lise Lien

Research Professor

Dr. Inger-Lise Lien (Social Anthropologist)

Norwegian Center for Violence and Traumatic Stress Studies (NKVTS)

Pb 181 Nydalen

NO-0409 Oslo

Norway

Tel: +4792403440

Manuscript Click here to access/download;Manuscript;Manuscript 3.docx

Unequal access to healthcare: a hysteresis condition?

Health workers' and Sub-Saharan African women's understanding of equal access to healthcare in Norway

Inger-Lise Lien[1*¶]

[1]Norwegian Centre for Violence and Traumatic Stress Studies, Oslo, Norway

[*]Corresponding author

¶ First author

E-mail: i.l.lien@nkvts.no (ILL)

[1]Norwegian Centre for Violence and Traumatic Stress Studies, P.O. Box 181, Nydalen, 0409 Oslo, Norway

Abstract

Aim and Method: This article discuss and analyses equal access to health care, based on interviews with health workers as well as Sub Saharan African Women. The study is conducted in Oslo, Norway, using a qualitative fieldwork research design. One hundred interviews were done with health workers and 55 interviews were done with Somali, Gambian and Eritrean women with the use of a semi structural guideline and purposeful sampling.

Results: The study found a mismatch in the conception of equal access to health care between health workers and the SSA-women. On the overall abstract level the health workers held that there was no equal access of health care for SSA-women, while SSA-women argued that there is equal access. However, at the practical level there was a great deal of match between the sides in conceptions of problems in the structure of health care like: little time available to explain and identify symptoms, navigation in the system, permeability like difficulties of getting referrals, adjudication by the health workers i.e.

Theory and analysis: The analysis applied Bourdieu's concepts of field, habitus and hysteresis, as well as candidacy theory and concluded that health workers and SSA-women based their experiences of health care within different types of habitus as their baseline for understanding. The SSA-women's habitus is in hysteresis with the current bio medical field, as they seem to base their understanding on earlier experiences from the home country.

Theoretical conclusion is that Bourdieu's theory is complementary to the candidacy theory and may function as a meta- theoretical framework for dimensions within the candidacy theory, thereby adding to a deeper understanding of reasons for match, mismatch and hysteresis that was expressed about equal access to health care by the two sides. Another finding was that the

SSA-women often suffer from unexplained pain, which leads them to seek health care from the religious realm within the traditional habitus, when they do not get satisfactory explanations from the bio medical field in Norway.

Introduction

In this article, equal access to healthcare is being analyzed on the basis of the perspectives of Sub-Saharan African women (SSA-women) from Gambia, Somalia, and Eritrea as well as the perspectives of health workers in Oslo. The article is based within a project called "Physical and psychological health care for girls and women with Female Genital Mutilation/Cutting (FGM/C)" that has been financed by the Norwegian Research Council and Norwegian Center for Violence and Traumatic Stress studies with the support from the Ministry of Justice.

Articles from the study have already been published showing that the organization and functioning of health care can lead to difficulties for SSA-women getting access to specialist mental health services due to silencing mechanisms, high standards of referral letters, long waiting lists as well as out pushing to the lower levels within the municipal services [1]. Another article [2] show that GPs are more likely to assess FGM/C as a possible cause of health problems when they have had pervious knowledge and experience with patients having FGM/C-related problems, and assess their own knowledge of FGM/C typology and medical codes as adequate. A third article [3] identified four cultural scenarios that influence SSA-women's intention to use FGM/C related services.

The aim of this particular article is to present attitudes held by the provider and receiver side of health care and to see how health workers and SSA-women assess equal access of health

care for women who come from countries practicing FGM/C. Research questions are: Is there consensus or a split between the two sides? Do the SSA-women find the health care services to be unjust and discriminating? Do SSA-women seek out alternatives in other countries or alternative treatment in their original home countries when they have health care needs, or are they satisfied with the health care that is provided by the biomedical health care system in Oslo?

In Norway, equal access to high-quality services is implemented in the Patient and User Rights Act, and it is argued that this should lead to promoting trust [4]. In a comparative study of trust of the healthcare system in several European countries, it was found that Norway in certain aspects scored relatively low compared to other European countries [5]. Other studies have shown that a general lack of trust in a society can impact the use of healthcare [6, 7], thereby leading to a negative spiral of both trust and the use of healthcare services. However, immigrants living in Norway, who seem to have worse living conditions than the majority of the population, still seem to have a high degree of trust in the political, social and healthcare systems [8]. On the other hand, studies also show differences in the use of emergency services (ER) and the GP [9]. Immigrants from Somalia use ER services more often than Ethiopean, Eritrean and Gambian immigrants [9, 10, 11]. Næss [12] argue that there is a lack of trust among the Somalis and argue that "cramped timeslots and a cut-to the case attitude among doctors" can be a poor foundation for trust", thereby placing reasons for distrust into the way that the services are organized.

This study found a split in attitudes between health care workers and the SSA-women that will be presented and analyzed in this article. At the practical level, however, there were agreements between the two parts about the functioning of the system. There were match and mismatch in the attitudes that require appropriate theoretical analytical tools in order to be

understood. The first choice of theory arose from the mismatch that was discovered at the more abstract level, where the concept of hysteresis used by Pierre Bourdieu [13] seemed to provide a pathway into a wider conceptual frame consisting of habitus and field. Complementary to the abstract meta-level analysis of Bourdieu [13,25,26), the candidacy theory developed by Dixson-Woods et al. [14] seemed to provide a very useful contribution for understanding access to health care through the seven dimensions at work in a "joint negotiation" of health care.

The article [14] is organized so that it will; 1) provide a background presentation of some theoretical discussions on equal access to health care; 2) introduce candidacy theory of health care negotiation as a meso-level theory, and 3) present an overall theoretical framework through the use of Bourdieu's theory of field, habitus and hysteresis. It will; 4) introduce the empirical findings arranged according to these two theories. Thereafter; 5) the health workers perspectives as well as the SSA-women's attitudes and problems with pain will be discussed and analyzed.

Equal access to healthcare: a theoretical background

According to Andersen et al. [15], access to healthcare is defined as "those dimensions which describe the potential and actual entry of a given population group to the health care delivery system." Access indicators can vary between countries due to how the services are organized. Aday and Andersen [16] as well as Goodhard and Smith [17] argue that equity of access exists when need rather than structural or individual factors determine who gains entry to the healthcare system. In a review of the research literature on access, Goodhard and Smith [17] criticize this research on access for being weak in terms of theoretical frameworks. They distinguish between a horizontal equity that requires equal treatment for individuals with equal

needs and vertical equity that requires that individuals with different needs receive different amounts and levels of services.

In a study from Italy, Glorioso and Subramanian [18] found inequity in health service utilization even though the country practices universal and egalitarian healthcare in principle. There was a significant amount of pro-rich inequity in the utilization of specialist services, but the use of primary care was found to be inequitable in favor of the less wealthy. Glorioso and Subramanian [18] argue that the benefits of prevention and early diagnosis are unequally distributed in favor of the wealthy. When health status is perceived as good, poorer groups tend to hold back their use of expensive or inefficient health services; that is, they limit their use of preventive care and postpone their access to curative care, creating the conditions for future worsening of their health status.

There are two sides to equal access of healthcare, namely a supply side and a demand side, and access to healthcare has mostly been studied from the supply side [17]. Ruger [19] focuses on the barriers that may reduce equal access to healthcare and chose a health capability perspective that focuses on users' resources. He argues that user groups must increase their knowledge so they can profit from the healthcare system. This way of thinking has been strengthened by others who are concerned with empowerment strategies and the degree of fit between the demand and supply sides.

Macintyre et al. [20] are concerned with the need for a systemic analysis that can inform policymakers about the "fit" between needs for healthcare and the receipt of healthcare. Within this connection, the researchers argue for several dimensions of healthcare, such as availability, affordability, and acceptability:

"Access, or empowerment to use health care services will only be achievable if all dimensions of access are addressed and both the health care system and individual perspectives are taken into account" [20:190]. This focus on "fit" and "gaps" between the provider and the receiver has led to an increasing interest in more closely examining the receiver side of healthcare, not only individually, but also from cultural and symbolic angles. New analytical concepts to capture resources on the demand/receiver side have been developed, such as empowerment, health literacy [21], and health capital [22].

Dixon Woods et al. [14] have attempted to develop concepts that bridge the two sides, such as candidacy: "candidacy describes the ways in which people's eligibility for medical attention and intervention is *jointly negotiated* between individuals and health services" [14]. Candidacy has seven dimensions that, according to the researchers, capture the characteristics at play during the joint negotiation [14,24]. These dimensions are 1. *identification*, which refers to users recognizing symptoms and health needs; 2. *navigation* by healthcare users, which requires an awareness of the services available; 3. *permeability* of services, implying accessibility of the services (may include competence to apply); 4. *appearances* at the health services (implying credibility, or ways of formulating the healthcare need in a credible way); 5. *adjudications*, which are judgments made by professionals from their interpretation of their clients' needs; 6. *offers and resistance* to reference by the patients as well as their reactions to offers of medication; and 7. *operating conditions*, which are locally specific influences on interactions between practitioners and patients.

The seven dimensions strongly emphasize the users' ability and work to present and formulate a claim for healthcare. On this receiver side of healthcare, there can also be resistance of the treatment provided. There can be difficulties in the beginning of developing a claim,

which are related to the identification of symptoms, knowledge about available services, and lack of competence in health issues or lack of economic ability to purchase the medication prescribed. On the provider side, there are adjudications and judgments that influence the end result of the healthcare negotiations and claims. The concept of negotiation captures many aspects of acting and communicating that occur before the healthcare-seeking process is initiated and claims for healthcare are made, as well as aspects belonging to the aftermath (like resistance to using medicine). Thereby, the concept of negotiation used in the theory is a very wide concept that includes works and claims developed by the patient during a very long process.

In analyzing the candidacy theory, van der Boor and White [23] discovered that in their review, there were more data on barriers to access than on negotiation of services. They appreciate the use of candidacy theory since it considers many relevant impacting factors but shows that there is an asymmetry in power and an enforcement of dominant values onto service users. They also found a rich experience of barriers to accessing and negotiating medical health services for their patient group, and the greatest problem during negotiation was language. Koehn [24] used the candidacy framework in a study of ethnic minority seniors' access to healthcare in Canada. According to Koehn, the first six dimensions in the theory deal with negotiation, but she argues that the seventh dimension represents the environmental context in which the negotiation occurs; thus, this dimension lies outside of negotiation [24].

Habitus and hysteresis in the medical field

In order to present a complementary theory as a contextual frame around the dimensions within candidacy theory, Bourdieu's theory of practice seems as an appropriate contribution [13,25,26]. Bourdieu operates on a meta-level making it possible to grasp

differences between groups of people at a wider and more abstract scale than that of the individual, through the use of the concept of field, habitus and hysteresis.

According to Bourdieu, a field is a place, or an overall structure where there is competition and inequality of power that affect the struggle for positions and access. Attached to the field is the habitus, an internalized structure that consists of codes, values, tastes, and distaste, schemata and memory traces, the result of experience, habit and socialization taken place in the field [25,26,27]. The habitus is embodied and can also be unconscious in the way that it is taken for granted. Through their practice and communication, persons may reveal their taste and values and emit signals on the basis of which people in other fields, with other habitus, can make distinctions and exclude them, and put them in a disadvantaged positions [25], or include them.

Hysteresis is a concept that Bourdieu [13] applies to identify mismatch between field and habitus, which occurs in situations of change and transformation. Hysteresis implies that something is "lagging behind". The field can change, so that the habitus is more in mismatch with the new field that has changed. When immigrants move to another country, their internalized habitus can be in mismatch with the new situation or structure in the new place which is a hysteresis condition due to their habitus lagging behind and being obsolete, and thereby it can lead to unfavorable decisions:

> "The presence of the past in this kind of false anticipation of the future performed by the habitus is, paradoxically, most clearly seen when the sense of the probable future is believed and when dispositions ill-adjusted to the objective changes because of a hysteresis effect are negatively sanctioned because the environment they encounter is too different from the one to which they are objectively adjusted [13, p 62].

The habitus concept has been criticized for being deterministic, and not sufficiently capable of describing change.[27,28:159].However, abstractions are necessary as tools for thought [29], and change is something that Bourdieu captures by his use of the concept of

hysteresis. A defense for using Bourdieu’s concepts of habitus is that it can analytically frame theories, like the candidacy theory, and thereby incorporate the understanding of differences and nuances of individual and group processes and negotiations, at the lower level. Due to migration, groups of people having different values and opinions are moving and spreading into different corners of the world, but all the same, grouping together into networks in the diaspora, having many similar habits and identities in spite of the differences between them. Migrants may create their own fields in the suburbs of cities where traits from the old habitus continues, or they may develop a new habitus. The habitus concept can capture the meaning making dimensions for groups of people that exist within these new field structures.

The biomedical system can be seen as a separate field. An old bio-medical habitus existing in peoples mind can also be in mismatch with a new developed bio-medical field, as has been described in a study from Denmark [30] using Bourdieu’s analytical concepts of hysteresis and habitus. The study found that the healthcare field over years had changed to an efficient and economically oriented market model. Danish family members at present, were expected to do more caring tasks than was expected in earlier times, like in the 1960s, when the healthcare and welfare systems were developed. The relatives of Danish stroke patients, having become adjusted to an earlier medical habitus version, therefore expected the current healthcare system to do most of the caring tasks rather than leave them to relatives or outsiders. This unfulfilled expectation led to irritation and disappointment. There was hysteresis between the habitus that the relatives of patients had adopted from earlier times and the way the medical field functions today or the present habitus in which the system has retracted from many caring tasks it used to perform, leaving them to relatives.

Bourdieu's concept of hysteresis shows that there is more to the interaction and communication between the healthcare system and patient groups than what the candidacy theory endorses alone. Behavior based on an older habitus that is mismatched with standards for candidacy held by health professionals today, who exercise their adjudication within a modern Western healthcare system, should not necessarily be understood as a failed individual strategic negotiation process. Rather, it may be a condition of hysteresis with standards and judgments of candidacy belonging to a medical field with its habitus that new patient groups have little knowledge of since their own habitus can lag behind or be attached to a different biomedical field.

In our analysis of the empirical findings in this study, dimensions within the candidacy theory are introduced and framed using the concept of field, habitus and hysteresis so that we can determine whether there is match, mismatch or hysteresis when it comes to understanding access to healthcare for women from immigrant communities, especially those from the SSA countries Somalia, Gambia, and Eritrea.

Method

This study is based on qualitative data collection in Oslo with participants on the supply and demand sides. One hundred interviews were conducted among health workers who were asked about their experiences with SSA-women. Participants were asked if these women received healthcare access, equal to that of the majority population. The interviews lasted for one to two hours each, and some participants were interviewed twice. The participants were general practitioners (24), psychologists and psychiatrists (9), gynecologists (4), neurologists (4), midwives (19), nurses (8), sexologists (3), and administrative health personnel (3). An assistant—a social anthropologist in her early 30s—conducted many of the interviews with the

health workers. The interviews were conducted in clinics, coffee shops close to the clinics, or private homes. The GPs were often very difficult to reach because they were busy and worked long hours. In spite of this, some agreed to be interviewed twice. The project leader—also a social anthropologist—took part in several of the interviews.

In addition, 55 SSA-women—20 Gambian, 20 Somalian, and 15 Eritrean women—were interviewed about their experiences with the Norwegian healthcare system.

The SSA-women had migrated to Norway either as refugees or as wives of migrants. None of them were born in Norway. They all come from the African continent and from traditions that cut girls, and they have in common being cut even though they practice different types of cutting and their problems and pains vary due to the different types of cutting. The World Health Organization (WHO) defines female genital mutilation/cutting (FGM/C) as "all procedures that involve partial or total removal of the external female genitalia, or other injury to the female genital organs for non-medical reasons." [31]

The average age of the Gambian women was 41, the Eritreans 46, and the Somalians 43. An Eritrean midwife helped to recruit the Eritrean women, mostly through her own Christian Tigrinya network. One Gambian woman, an activist and a member of the project's user group panel, took part in the project as a gate opener in the Gambian community and conducted some interviews with the Gambians herself. A Somali midwife in her 50s was willing to assist in recruiting and interviewing Somali participants, making use of her huge network in the Oslo area. The anthropologist (in her 60s) did interviews either with an assistant or alone with women within all three ethnic groups. Since she had done fieldwork among Somalis and Gambians in earlier projects [32,33,34,35], confidence and trust have been established over the years. All interviewees received both oral and written information about the project and signed a letter of

consent. The interviews lasted approximately two hours, and several of the participants were interviewed twice. Interviews were not audio recorded since the participants were very negative and skeptical about recording. Handwritten notes were taken during the interviews and later typed into the computer and analyzed. Using contacts made through acquaintances and key participants is a qualitative method commonly used by social anthropologists, and it implies borrowing the trust the assistants have in the community. It is a purposive network sampling procedure. The Ethical Review Board (REK) in Norway approved this study on September 12, 2017.

Findings

Health workers' habitus and perspectives on equal access to healthcare for SSA-women

All health workers were asked if SSA-women receive equal access to healthcare. The immediate response was negative. However, healthcare workers continued their reflections by saying that in principle all immigrants should have equal access to healthcare, but in reality they do not receive it. They argued that the medical system is hierarchical and bureaucratic and meets clients on its own terms rather than on those of the patient. The system is to blame for being rigid and not giving patients enough time, competence, and understanding. Some participants said that the system discriminates between those who have resources and those who do not. Others said that doctors and nurses are too impatient, jumps to conclusions, and do not provide enough consideration for patients who do not speak the language and have little health competence. Health workers talk behind patients' backs and stigmatize them; therefore, women with immigrant backgrounds like the SSA-women do not receive equal access to healthcare. Below are some of the responses:

> "No, they get three-quarter services. The immigrant women must wait longer because they are more difficult to treat. They change their GP a lot, and I think it is because they do not get the treatment they want. Some do not show up for their appointment or do not pay, and this means they are not wanted as patients." (GP, female, 63)

> "In Norway you have to stand up for your rights, and they do not know their rights." (GP, female, 52)

> "No, there is lack of cultural understanding in the health services." (Midwife, 61)

> "The doctors don't give an offer that fits with the background of the patient." (GP, female, 45)

> "Sometimes seven people arrive for consultancy when there should only be one patient. There are lots of prejudice against them, but some of the prejudice is real. You have to be realistic." (GP, female 50)

> "They express their illness in a diffuse way, and it is difficult to understand what they say." (GP, female, 43)

> "There is no equal access. Our system is complicated, and it is difficult to navigate. The use of interpreters makes the situation difficult also. When we have consultancy with an interpreter in the room, it takes the double amount of time." (Midwife, 58)

> "No, they are discriminated at all levels of the society." (Sexologist, 48)

Arguments from health workers explaining why there is not equal access to healthcare in the Norwegian health system are listed above and below. Some of the health workers blamed the overall system in terms of how the biomedical field functions structurally, hierarchically, and bureaucratically. Second, health workers put the blame for unequal access on attitudes held by health workers (i.e., on their own habitus). Third, health workers put blame on the SSA-women's ways of behaving and thinking, their lack of knowledge about the biomedical system in Norway, their lack of competence and education when it comes to body and health, their lack of knowledge about their rights. This is health workers conceptualization (habitus) of SSA-

women's communication of needs that can be categorized and summarized as the health workers' "ideas about SSA-women's habitus".

(S1 Table 1)

The morality within the biomedical habitus of health workers seems to be based on a human rights conception, and health workers often complained that SSA-women did not know their human rights and therefore did not claim their rights. From health workers perspective they could see the mismatch between their own thinking and that of the SSA-women, who may reason and behave in another way than what they expected – ways of behaving and thinking that we have categorized as lying within the SSA-women's habitus

Health workers think that SSA-women have problems with navigation, permeability, and appearances that can be linked to the fact that they are poor, refugees and migrants who have other ways of diagnosing their illnesses (i.e., according to Bourdieu's terminology: a different medical habitus). As candidates for healthcare, they are seen as lacking competence and system understanding, and due to language problems they are seen as communicating their healthcare needs poorly. The candidacy dimensions developed by Dixon-Woods et al.[14] are formal descriptions, so that we can fill them with substantive contents. The substantive contents we have filled in the left side of the table, and when it comes to adjudications, there is both stereotypes, underestimation, stigma and rejection as interpreted by the health workers themselves, and when it comes to permeability there is resistance towards referrals of these women in the system.

When health workers discuss the substantive content of the navigation dimension of the SSA-women's habitus, they fill it with weak system competence, incorrect use of the system, weak networks and not knowing their rights so that they can make adequate health care claims. This indicates that the health workers can assess the hysteresis that Bourdieu has described, when

there is a mismatch between the habitus and the requirements of the field. The overall conclusion of the health workers is that there is not equal access to healthcare because of these system factors, the mismatch between health workers' ways of thinking, and the SSA-women's ways of acting and thinking –their habitus.

SSA-women's conceptions of equal access to healthcare

When asked if they receive equal access to healthcare, the SSA-women immediately answered affirmatively and then added that they are very satisfied: "I have 100 % confidence in the Norwegian health care system" (Eritrean, 55). "The Norwegian health system is the best; I only have good experience" (Somali, 42). "They don't say that you are African and we have to take Norwegians first. No, no, no. It is equal. We get the same treatment and priority as the Norwegians. I am satisfied" (Eritrean, 53). "I have had two GPs. The first one I could have married, and the second one could have been my sister" (Gambian, 48).

As an example of how good the Norwegian healthcare system is and how generous the GPs are, a Gambian woman explained that when she divorced her husband, the GP even came to her home, talked with her for a long time, and helped her to apply for social security money, and she got sick leave for many weeks. She said that she loved the GP. Others said, "The Norwegian health system is first class" (Gambian, 36) and "The Norwegian system has good hygiene, good medicines, and nice doctors" (Gambian, 23). However, two interviewees out of 55 had heard about discrimination against immigrants, though they had not experienced it themselves. One woman said that she thought there was discrimination in the healthcare system and that she had experienced it herself. These comments were exceptions in the material. Mostly, the interviewees talked very positively about the healthcare system with a great deal of respect and trust.

Principally and as a moral imperative, they expressed a deep trust and confidence in the health system in Norway and especially in their GPs.

However, there is another level in the SSA-women's analysis of the healthcare system. After first having stated their very positive trust that they received equal access to healthcare, their confidence in and respect for the Norwegian healthcare system and its healthcare workers, there were issues about which the SSA-women complained. "The waiting time for consultancies is too long" (Eritrean, 52). "The GP does not ask us about FGM/C and does not understand the problems that this procedure cause" (Eritrean, 52). "If the doctors examine us in our private areas, they do not discover that we are cut or mention that we are cut" (Gambian, 50). "They do not understand us who are circumcised" (Gambian, 35). "Some of the GPs do not have a lot of competence because they use the Google a lot. I could treat myself by investigating the Google. That is what the doctors do" (Somali, 48). Another issue is that the GPs seem insecure and ask the patients what is wrong with them instead of telling the patient what is wrong: "In Africa, it is the doctors who decide. In Africa, doctors take responsibility. In Norway, when you are ill, doctors want to collaborate with you. It is as if they do not care about you. They ask you, 'What medicine do you want?'" (Somali, 59).

There was a general attitude among the Eritrean, Gambian, and Somali women that they found the health system to be fair and good. This goes for all the three ethnic groups. However, they found that doctors were sometimes insecure about their own competence, consulted with books or the internet, and asked the patients questions, so the patients thought the doctors did not have sufficient knowledge. The women's understanding and assessment of the healthcare system also seem to be based on the fact that they compared the system in Norway not against

ideologically based principles about rights grounded in policy documents and moral principles, but rather against the system in their original homeland, and they all concluded that the Norwegian system is better. In this way their biomedical habitus – based in the home country, is used as a baseline to assess the biomedical system in Norway. The women do not prefer the healthcare system in their home country. They do not call relatives and friends in other countries to give them advice when they are ill, nor do they express a wish to seek help from medical systems in other European countries. However, on another level they have found deficiencies and weaknesses in the Norwegian medical system that they mentioned. Below is a table showing a list of positive attitudes as well as complaints that were expressed during the interviews.

The candidacy theory is used somewhat different here than what it is supposed to, as presented in the table below. The dimensions described is usually thought to be based on the usergoups claim during negotiation. Here it is reversed, it is the SSA-women's thoughts about health workers identification, appearances, permeability and adjudication of services that are assessed.

(S2 Table 2)

Among the most mentioned complaints is that the women are not understood when it comes to FGM/C and that FGM/C is not mentioned. "The experience with the healthcare system has been good but lacking in regards to conversations around FGM/C. It seems as a taboo here as in the Gambia. It was never mentioned" (Gambia, 48). "I felt very much discriminated by the doctor. I have no good communication with him. They just write and write and do not have the time, just 10 minutes, and then they take a lot of money from you" (Somali, 55).

This is an organizational and structural analysis conducted by the women that focuses on the time available and ways of making diagnoses and referrals. Here we find that there is

match between the conceptions of health workers and the SSA-women. However, patient involvement that is usually seen as a democratic right of patients to take part in decision making is not necessarily viewed as something positive and in line with the women's habitus and expectations of the doctor. Instead, it can be viewed as a weakness of the system, which is a condition of hysteresis between different habitus.

Unexplained pain

Another issue that deals with health workers' identification of symptoms is that many women have pain that has not been diagnosed. Eritrean, Somali, and Gambian women mentioned this often. "How come that you have pain, day in and day out, and the GP tells you that there is nothing wrong with you?"(Somali, 55).

> "My pains kill my brain." (Gambian, 54)
>
> "I do not understand that when I go to a GP and tell him that I have pain, he explains that I am not ill. Then I think I must have pain the rest of my life. I must just get used to pain. But I also think that this pain is not normal, that the GP is stupid, that there is a lot the doctors do not know." (Somali, 58)
>
> "I have confidence in the health system and do not think they discriminate. But they must do more examinations and take people more serious when they have pain. They cannot just say 'Take it easy; everything is fine.'" (Gambian, 48)

Out of 53 women who discussed their pain during the interviews, 36 women said that they suffer from pain and illnesses. The pains are often around the reproductive area as well as in the legs, back, and stomach. Eleven women suffered from pain that had received a diagnosis. Twenty-five women said that they suffered from pain that was undiagnosed and unexplained by the GP.

Several of the Gambian women thought that their chronic pain was due to the cutting operation done many years previous. Seven Gambian women out of 20 (35%) argued that they had problems in their reproductive system as well as pains and dryness during sex, and according to some of them, this could be caused by female genital mutilation. They linked their pain as well as their depression to the effects of having been cut many years back. “I go to the doctor all the time, and one doctor called it referral pain due to FGM” (Gambian, 48). Another woman linked her urinary infections to the cutting operation: “When I sit in a bus stop and it is cold, I will get urinary infection. It is because my labia has been removed during circumcision and do not protect me from cold. I get antibiotics. It is pain when urinating, and then it is difficult with sex and you feel ill all the time. Then you become depressed” (Gambian, 40).

When the biomedical healthcare system does not provide help for psychological and physical suffering and pain, women can turn to spiritual healing rather than seek help from biomedical systems in other countries. Many of the women explained that they did not believe in spiritual healing and had not used imams, priests, or marabous to help them when they were ill, but many had contacted religious men in their previous home country to help them. Amulets, Qur’anic verses, beans, and holy water were sent from abroad. Telephone calls were made to spiritual healers, and holy men (called “marabou” in Gambia) performed ritual slaughtering of a goat or chicken, usually in Africa after orders were sent via telephone or SMS. It seems like spiritual healing is particularly effective when it comes to mental issues. Some interviewees said that they felt much stronger and had more hope of recovery because the spiritual healers would pray to God and try to improve their health or destiny via different rituals performed in the original country and in Norway. There are certain streets in Oslo where spiritual healers can be found. Explanations for suffering could be the evil eye, witchcraft, or jealousy that other people

feel toward them, pushing individuals to seek protection and to remove black magic that could have been put on them or djinns that are troubling them, causing them to feel pain and receive ill fortune.

Each of the three groups has its own tradition that includes an inbuilt healthcare system that is connected to the religious tradition. Eritreans are connected to the Pentecostal church and believe in praying and laying on of hands. Every Friday in the Pentecostal church there are prayers for those who are ill and have pains. “When you have psychological problems, you go to the church and pray” (Eritrean, 55).

The Somalis also seek healthcare within their religion, and they distinguished between going to the imam, a religious healer, and then going to an Islamic conference outside the country. Different imams and/or healers are believed to have more ability to cure than others.

> “I have a lots of pain. Therefore, I go to the Mosque and pray.” (Somali, 48)

> “If a Somali woman has a psychological problem or pain, she will not go to a psychologist, because that means she is nuts. She would rather go to an imam that tells her that there is a djinn within her. He will then try to remove the djinn.” (Somali, 31)

> “To go to the mosque is first choice. The second choice is to go to Sweden in the summer where there is a big Islamic conference. People go there to get healed. Swedish imams, and especially English imams, are seen as more competent than the Norwegian Imams. The imams do not demand payment, but people must pay according to their ability and desire.” (Somali, 35)

The SSA-women thereby have alternatives based within their traditional habitus to be used in their navigation for healthcare. This is an extension of their medical field and habitus that includes more options for healthcare and health treatment than what the public biomedical healthcare system can provide. Some of the SSA-women seem to slide into religious healing

when they feel that the biomedical system does not provide a sufficient cure when they have pain and when they are not referred further.

According to Kleinman [36], physicians have a narrow focus on diagnosis and treatment (disease), which leads them to exclude the patient's experience (illness) from their consideration. When medical doctors do not manage to provide a diagnosis or treatment, patients may search for alternatives within the spiritual realm, implying that their management of suffering and their illness widens their medical field and habitus, where spiritual healing becomes an important part. Alternatives like these are often seen as illegitimate by biomedical experts and represent a hysteresis condition to the biomedical field. However, patients from an African background may use these alternatives in combination with the biomedical system in order to get cured. We can regard these alternatives as complementary to the biomedical system, even though it functions according to non-scientific criteria and thinking and is often part of an older habitus or the habitus from the more spiritual realm.

Hysteresis and match in the conceptions of equal access to healthcare

Health workers have an *internal position* in the biomedical system from which they can observe the interaction that takes place between health workers at different levels and between the patients and the system. They can hear arguments and comments that are passed during both formal and informal circumstances that deal with receiving, treating, or excluding patients. In addition, their job positions are based on medical and health studies in which a scientifically based healthcare, human rights, and medical ethics are included in the curriculum. From this background as well as from experiences and observations in practical life, health worker participants in this study concluded that there is not equal access to healthcare for women from

SSA backgrounds. These health workers presented arguments that put the blame for inequity on the system, how it is organized, the attitudes of health workers, as well as the SSA-women's own behavior and forms of communication—that is, on their habitus, where health workers have observed some mismatch in expectations. The health workers are disappointed with the system since they may have an ideologically based high expectation of justice from where they judge equal access to healthcare as not fulfilled according to their own observations and assessments.

The SSA-women have an *external position* from which they seek access to the system. At the principal level, the SSA-women seem to trust the overall system when it comes to its will to diagnose, refer, treat, and care for their health. They seem not to have an expectation based on the formulation of rights from where they measure their achievements to healthcare, making them disappointed with what they receive. In fact, they seem to think that the system and the healthcare workers provide them with a kind of help they would not have received in their original home country. In this there seems to be a mismatch and hysteresis between their understanding of the situation and the health workers' conceptions of the situation. The SSA-women have both a positive experience of the medical health system as well as an expectation for help that seems to be more or less fulfilled since they have a background from another medical field in the African continent that can provide less medical healthcare than what they get in Norway. There is a clear gap, mismatch and hysteresis between the demand and supply sides of the medical system when it comes to an overall understanding of equal access to healthcare for SSA-women. Having an outsider perspective, the SSA-women can be ignorant about discriminatory factors on the supply side, because exclusion factors are invisible to them and very few women reported that they have experienced discrimination by health workers when they have been in need.

However, on *the practical level* there is a match between the two parties' understanding when it comes to the perceived weaknesses of the system, such as available time, shortage of opportunities at the GP's office to explain healthcare needs, lack of the use of interpreters and problems of cultural and language communication and understanding during consultancies, and lack of referrals to specialists. There is a feeling among some of the SSA-women that there can be barriers to being referred further, which is confirmed by the health workers' conception. The parties agree that there are some unfortunate deficiencies within the system, even though the two parties also stressed different aspects, such as the competence issue where health workers put blame on the SSA-women for their lack of health competence while several of the SSA-women think that some doctors must be incompetent when they do not find the reasons for their pains and do not have competence and willingness to discuss FGM/C. When the SSA-women fail to receive a diagnosis, are disappointed with a diagnosis, or are not cured, they sometimes turn to spiritual healing, which we can describe as returning to their traditional habitus. There is obviously a mismatch and hysteresis between the parties' understanding of the causes of unequal access to healthcare.

Conclusion

The hysteresis apparent in the conceptions of equal access to healthcare between the two sides has mostly been analyzed in terms of Bourdieu's concepts of habitus and field. In addition, Dixon Woods' theory of candidacy has been presented in which she uses "joint negotiation" to explain the driving forces of access to healthcare.

However, the concept of negotiation, with the attached dimensions, used within candidacy theory, is broad and goes further than the negotiation taking place in a consultancy room, as described by Sandman [37] and Sandman and Munthe [38], were people bring relevant

issues at the table in order to obtain and negotiate their aim in rational and argumentative ways. In the candidacy theory the negotiation process includes events and happenings, which took place long before the first meeting with health professionals. In this way, the concept of negotiation transgresses into the reality of the habitus of the patient groups, on the supply side. By doing so, it touches upon very relevant factors within the habitus, but without being able to frame it analytically through a coherent structure of internalized codes, understandings, and values obtained both consciously and unconsciously within a long socialization process. These frames, in this study called “habitus” can be diverse due to the different people and fields, and they can match or be in mismatch with different fields, structures or people involved within health care. Candidacy theory as an analytical instrument can dig into the habitus of different groups, but to a lesser degree frame a coherent habitus.

Through the use of the negotiation conception with all seven elements included, the importance of the demand side in order to get access seems to outweigh the importance of the supply side. In reality, it is the adjudication done by the health workers that in the end provides the ultimate power to define the tipping points for the candidates’ entry. This is what interviews with health workers in our study stressed. These workers have been aware of their power as well as the SSA-women’s lack of power and knowledge about rights. In the candidacy theory, the blame for unequal access to healthcare is to a great extent put on the demand side even though responsibility is theoretically thought to lie in the middle as a “joint negotiation.”

Power relations within healthcare are not necessarily visible to user groups who are new to a country and have little competence and scientific knowledge about the healthcare system. If their communication about healthcare needs is based within an old habitus from another situation with other ways of formulating symptoms and needs, this can lead to hysteresis between existing

criteria for access and people’s behavior and bodily signals. Medical health personnel can, on the basis of the received signals from the habitus that other people carry, open and close doors for access; thereby, they may also be able to observe the hysteresis that exists between different habitus and the habitus of the medical field. This observation is based from within and is a benefit for assessing equal access to healthcare that the patients who try to obtain access do not necessarily have.

In recent years there has been a development in the medical sector away from paternalism towards a more democratic healthcare approach. There is an expectation that most patients have a high level of education and knowledge about health issues—so much so that they are welcome to have a say when it comes to medication and healthcare.

Complaints from the SSA-women directed towards the health care system has dealt with structural and organizational matters. In addition SSA-women have focused on the lack of competence when it comes to FGM/C. Many of them suffer from unexplained pain that needs to be cured. Not only should SSA-women get an offer for reproductive and sexual counselling, but they should also get an offer for pain management and treatment.

In a situation where new patient groups have less schooling and scientific knowledge about health issues and less knowledge about the system and their rights, the biomedical field is, to a greater extent than before, in the way it is organized and functions, in hysteresis with the new reality involving new patient groups. This can have a negative effect on the new patient groups’ equal access to healthcare, as this study demonstrates. Unequal access to health care can also be something that new user groups may not at all see, or be aware of, as this study has also shown.

Acknowledgments

I would like to thank Cecilie Knagenhjelm Hertzberg, Neneh Bojang, Safia Abdi Hasse, Sara Khasay and Sara Zorica Mitic for their contribution in the data collection process. I will thank Health Forum for Women in Oslo for access to some informants. I will also thank Mai Mahgoub Ziayada, R. Elise Johansen and Marianne Opaas at NKVTS for their support and contribution to the project, as well as professor Rune Halvorsen for his reflections, contribution to new literature as well as comments on the theoretical analysis, particularly to Bourdieu's concept of habitus.

References

1. Lien IL & Hertzberg CK. A system analysis of the mental health services in Norway and its availability to women with female genital mutilation. 2020. PLOS ONE. ISSN 1932-6203. 1- 19 . doi: https://doi.org/10.1371/journal.pone.0241194

2. Ziyada MM, Johansen REB, Berthelsen M, Lien IL, Bendiksen B. The implication of training and knowledge on female genital cutting (FGC) for the proactive assessment of FGC as possible cause of health problems: A cross-sectional survey among general practitioners in Norway. BMC Health Services Research 2021, submitted

3. Ziyada, MM, Lien IL.& Johansen R EB. Sexual norms and the intention to use healthcare services related to female genital cutting: A qualitative study among Somali and Sudanese women in Norway. 2020. PLOS ONE. doi: https//doi.org/10.1371/journal.pone.0233440

4. Helsedirektoratet. Pasient- og brukerrettighetsloven LOV-1999-07-02-63. https://lovdata.no/dokument/NL/lov/1999-07-02-63?q=Lik%20rett%20til%20helsehjelp

5. Holmboe O, Iversen HH, Sjetne IS, Skudal, KE. Commonwealth Fundundersøkelsen 2011 blant utvalgte pasientgrupper: Resultater fra en komparativ undersøkelse i 11 land. Rapport fra Kunnskapssenteret nr 18-2011. Oslo: Nasjonalt kunnskapssenter for helsetjenesten, 2011.https://www.fhi.no/globalassets/dokumenterfiler/rapporter/2011/rapport_2011_18_commonwealth_2011_rettetv2.pdf

6. Feldmann TC, Jozien M, Bensing; Ruijter Arie de and Hennie R. Boeije (2007) Afghan refugees and their general practitioners in The Netherlands: to trust or not to trust? Socioloogy of Health & Illness, Vol 29 No 4 2007 Doi: 10.1111/j.1467-9566.2007.01005.x

7. Ku L. and Matani S. Left out: Immigrants' Access to health Care and insurance. Health Affairs. 2001. Volume 20, no 1

8. Barstad, A. and Sandvik L. Deltaking, støtte, tillit og tilhørighet. En analyse av ulikhet i sosiale relasjoner med utgangspunkt i levekårsundersøkelsene . Rapport SSB 2015/51

9. Diaz E, Kumar BN. Differential utilization of primary health care services among older immigrants and Norwegians: a register-based comparative study in Norway. BMC Health Serv Res. 2014;14:623.

10. Diaz E, Calderon-Larranaga A, Prado-Torres A, Poblador-Plou B, Gimeno-Feliu LA. How do immigrants use primary health care services? A register-based study in Norway. Eur J Pub Health. 2015;25(1):72–8. 15.

11. Diaz E, Mbanya VN, Gele A, and Kumar B. Differences in primary health care use among sub-Saharan African immigrants in Norway: a register-based study. BMC Health Services Research 2017 17:509 DOI 10.1186/s12913-017-2404-z

12. Næss, A. Trust, cultural health capital, and immigrants' health care integration in Norway. Sociology, 53(2), 297-313, 12

13. Bourdieu, P. (1990). The Logic of Practice. Cambridge University Press. Cambridge

14. Dixon-Woods M, Cavers D, Agarwal S, Annandale E, Arthur A, Harvey J, Hsu R, Katbamna S, Olsen R, Smith L, Riley R and Sutton AJ. Conducting a critical interpretive synthesis of the literature on access to healthcare by vulnerable groups BMC Medical Research Methodology 2006, 6:35 page 7, doi:10.1186/1471-2288-6-35

15. Andersen RM, McCutcheon A, Aday LA, Chiu GY, and Bell R. Health Services Research 1983: 18:1 (p.51)

16. Aday LA & Andersen RM. The National Profile of Access to Medical Care: where Do We Stand? American Journal of Public Health 1984; Vol.74, NO.12

17. Goodhard M and Smith P. Equity of access to health care services: Theory and evidence from the UK. Social Science and Medicine 53 (2001) 1149-1162

18. Glorioso V. and Subramanian SV. Equity in Access to Health Care Services in Italy. HSR: Health Services Research49:3 (June 2014) DOI: 10.1111/1475-6773.12128

19. Ruger JP. Rethinking equal access: Agency Quality and norms. Global Public Health, 2007. 2:1, 78-96. https://doi.org/10.1080/17441690600911480

20. Macintyre DI, Thiede, M, Birch S. Access as a policy-relevant concept in low- and middle-income countries. Health Economics, Policy and Law (2009), 4: 179–193 p. 190, doi:10.1017/S1744133109004836

21. Nutbeam D. The evolving concept of health literacy. Social Science & Medicine 67. 2008. 2072-2078

22. Shim JK. Cultural health capital: a theoretical approach to understanding health care interactions and the dynamics of unequal treatment. J Health Soc Behav. 2010;51(1): 1–15.

23. van der Boor C F & White R. Barriers to Accessing and Negotiating Mental Health Services in Asylum Seeking and Refugee Populations: The Application of the Candidacy Framework. Journal of Immigrant and Minority Health.2020. 22:156-17 https://doi.org/10.1007/s10903-019-00929-y

24. Koehn S. Negotiating candidacy: ethnic minority seniors' access to care. Ageing & Society 29, 2009, 585-608

25. Bourdieu P. Distinction: a Social Critique of the judgement of taste. 1984. London: Routledge & Kegan Paul

26. Bourdieu P. The weight of the world: social suffering in contemporary society. 1999. Oxoford: Polity.

27. O'Reilley K. International migration and social theory. 2012. Basingstoke, Palgrave Macmillan

28. Jenkins R. Pierre Boudieu.2002. Rev edn. London: Routledge

29. O'Neill O, Towards Justice and Virtue.1996. Cambridge University Press https://doi.org/10.1017/CBO9780511621239

30. Roenn-Smidt H, Shim J.K, Larsen K & Hindhede A.L (2020) Hysteresis – or the mismatch of expectations and possibilities among relatives in a transforming health care system. Health Sociology Review, 29:1, 32-44, DOi: 10.1080/14461242.2019.1704425

31. World Health Organization. Eliminating female gential mutilation: An interagency statement. Geneva, Switzerland: WHO; 2008.

32. Lien IL & Schultz JH. Internalizing Knowledge and Changing Attitudes to Female Genital Cutting/Mutilation. Obstetrics and Gynecology International, 2013 (467028), 1-10. doi:10.1155/2013/467028

33. Lien IL & Schultz JH. Interpreting Signs of Female Genital Mutilation within a Risky Legal Framework. International Journal of Law, Policy and the Family, 2014. 28(2), 194-211. doi:10.1093/lawfam/ebu002

34. Schultz JH and Lien IL. Cultural protection against traumatic stress: traditional support of children exposed to the ritual of female genital cutting. International Journal of Women's Health, 2014(6), 207-219. doi:10.2147/IJWH.S51988

35. Lien IL, The perspectives of Gambian men on the sexuality of cut and uncut women. Sexualities. 2017. 20(5-6), 521-534. doi:10.1177/1363460716675142

36. Kleinman A. Patients and healers in the context of culture. An exploration of the borderland between anthropology, medicine, and psychiatry. 1980.Berkeley, Los Angeles, and London, University of California Press, 1980

37. Sandman L , The Concept of Negotiation in Shared Decision Making. Health Care Analysis 2009 17:236-243. DOI 10.1007/s10728-008-0103-y

38. Sandman L and Munthe C. Shared decision making, Paternalism and Patient Choice. Health Care Analysis. 2010 18:60-84. DOI 10.1007/s10728-008-0108-6

Supporting information

S1 Table.pdf

S2 Table.pdf

S3 Table.pdf

Click here to access/download
Supporting Information
S1 Table 1.pdf

Click here to access/download
Supporting Information
S 2 _Table 2.pdf

Click here to access/download
Supporting Information
S3 Table 3 Health worker Informants .pdf

Click here to access/download

Supporting Information

S1 appendix this is interview guide.pdf
